# The abbreviated form of the Brief Cognitive Battery in the diagnosis of dementia in Alzheimer’s disease

**DOI:** 10.1590/S1980-57642009DN30400011

**Published:** 2009

**Authors:** Stephanie Castro, Antonio Eduardo Damin, Cláudia Sellitto Porto, Paulo Caramelli, Ricardo Nitrini

**Affiliations:** 1 MD, Undergraduate Student, Departments of Neurology, School of Medicine, University of São Paulo, São Paulo SP, Brazil; 2 PhD, MD, Departments of Neurology, School of Medicine, University of São Paulo, São Paulo SP, Brazil; 3 PhD, Departments of Neurology, School of Medicine, University of São Paulo, São Paulo SP, Brazil; 4 MD, Department of Neurology, Federal University of Minas Gerais, Belo Horizonte MG, Brazil.

**Keywords:** Alzheimer’s disease, dementia, diagnosis, education, brief cognitive battery, neuropsychological tests

## Abstract

The Brief Cognitive Battery (BCB) developed by our group for cognitive assessment of low educated individuals has also shown to be highly accurate in diagnosing dementia of individuals with medium or high levels of education, making it a useful tool for populations with heterogeneous educational background. The application of BCB takes around eight minutes, a rather long period for a screening test.

**Objectives:**

Our aim was to evaluate whether the exclusion of items of the BCB could reduce its application time without losing accuracy.

**Methods:**

Patients with Alzheimer’s disease with mild or moderate dementia (N=20), and 30 control subjects were submitted to an abbreviated version of the BCB in which the clock drawing test was not included as an interference test for the delayed recall test. Data from another 22 control individuals who were submitted to the original BCB in another study were also included for comparison. A mathematical formula was employed to compare the two versions of the BCB. Descriptive statistics and ROC (receiver operator characteristic) curves were used (alpha=0.05).

**Results:**

Using the abbreviated version, the delayed recall test also had high accuracy in diagnosing dementia and the mathematical formula results did not differ to those obtained using the original version, while mean time was reduced by 2 minutes and 37 seconds.

**Conclusions:**

This abbreviated form of the BCB is a potentially valuable tool for screening dementia in population studies as well as in busy clinical practices in countries with heterogeneous educational backgrounds.

## Abstract (pt)

A Bateria Cognitiva Breve (BCB), desenvolvida pelo nosso grupo para a avaliação cognitiva de indivíduos de baixa escolaridade, tem se mostrado também bastante precisa no diagnóstico de demência em indivíduos com escolaridade média ou alta, tornando-se uma ferramenta útil para populações com heterogeneidade educacional. A aplicação da BCB demora cerca de oito minutos, um tempo bastante longo para um teste de rastreio cognitivo.

**Objetivos:**

Avaliar se a exclusão de itens da BCB pode reduzir o tempo de aplicação, sem perder precisão.

**Métodos:**

Pacientes com doença de Alzheimer com demência leve ou moderada (N=20) e 30 indivíduos controle foram submetidos a uma versão abreviada da BCB na qual o teste do desenho do relógio não foi incluído como um teste de interferência para o teste de recordação tardia. Dados de outros 22 indivíduos controle que haviam sido submetidos à BCB original em um outro estudo foram incluídos para comparação. Uma fórmula matemática também foi empregada para comparação entre as duas versões da BCB. Estatísticas descritivas e curvas ROC (receiver operator characteristic) foram utilizadas e admitiu-se alfa=0,05.

**Resultados:**

Com a versão abreviada, o teste de recordação tardia também teve alta acurácia no diagnóstico de demência, os resultados das fórmulas matemáticas não foram diferentes daqueles obtidos com a versão original, e o tempo médio foi reduzido em 2 minutos e 37 segundos.

**Conclusões:**

Esta forma abreviada da BCB é uma ferramenta potencialmente útil para o rastreio de demência em estudos populacionais, bem como para o emprego em prática clínica em países em que há grande heterogeneidade educacional.

Alzheimer’s disease is the leading cause of dementia worldwide, accounting for 54% (as the sole cause) to 69% (when associated with cerebrovascular disease) of the cases in a Brazilian population study.^1^ The most used tests for functional and cognitive evaluation for the diagnosis of Alzheimer’s disease (AD) in Brazil^2^ are the Mini Mental State Examination (MMSE)^3,4^ and Functional Activities Questionnaire (FAQ).^5^ With the exception of the FAQ, almost all the other tests are influenced by the level of schooling of the patient, since they involve reading and oral understanding skills, and their scores change depending on level of schooling.

In the last 15 years, our group has been working on the Brief Cognitive Battery, a battery designed for the evaluation of low educated individuals. It is constituted by the identification and naming of simple drawings of 10 common objects, followed by the incidental memory of these objects. Subsequently, the drawings are presented on two more occasions, followed each time by the recall of the objects, to obtain the scores of immediate memory and the number of learned or encoded items, called the learning score. This is followed by an interference phase comprising a category fluency test (animals in one minute) and the clock-drawing test.^6^ After this interference, (free) delayed recall and recognition of the 10 objects amongst 20 drawings (with 10 distracters) are evaluated.

The BCB has been shown not to be heavily influenced by education in several studies,^8-10^ and a proposal was made to change its name to the Brief Cognitive Battery – Education (BCB-Edu)^10^ so as to emphasize the low influence of schooling. The battery is particularly useful in populations with heterogeneous levels of schooling, a common occurrence in developing countries, principally in the elderly. Another characteristic of this battery is that its application method does not need to be changed when evaluating illiterates or highly educated individuals.

The application of the BCB takes eight minutes on average,^10^ making it rather long to be used as a screening tool in population studies. It would be valuable if the BCB could be quicker for use in population studies and in primary service. To achieve this, items of the BCB should be excluded.

Results of a recent study showed that a mathematical formula including four items, three items from the BCB – learning, delayed recall and verbal fluency scores – and years of schooling, have high accuracy for the diagnosis of dementia in a sample of AD patients and controls.^11^ As the visual perception and nomination of the drawings, incidental memory and immediate memory are necessary for learning and delayed recall tests, they cannot be excluded. Since the clock drawing test, which evaluates executive skills, was reported to be difficult to perform by non-demented illiterate patients,^8^ and the test of recognition of ten figures does not demonstrate high specificity or sensitivity in diagnosing dementia,^10^ we decided to apply the BCB without these two items.

Our aims in this study were to investigate whether the BCB can be reduced by eliminating these two items, without loss of accuracy in the differential diagnosis between normal individuals and AD patients with mild or moderate dementia, as well as to verify whether this abbreviated form of the BCB is similar to the standard BCB regarding other characteristics.

## Methods

### Subjects

Fifty individuals were recruited, 20 AD patients according to the NINCDS-ADRDA criteria, with mild or moderate dementia according to the DSM-III-R criteria,^12,13^ and 30 individuals without cognitive disturbances, with CDR (Clinical Dementia Rating)^14,15^ of zero. These volunteers were included as the control group (Control group I). Exclusion criteria, both for patient and control groups, were: non compensated systemic diseases or depression, visual or hearing problems that could interfere with the cognitive performance, past or current history of alcoholism, as well as other types of dementia besides AD. For the control group, use of medications that could interfere with cognitive performance was also part of the exclusion criteria.

Data from another 22 control individuals (control group II) who were submitted to the original BCB in another study^10^ were also analyzed to allow comparisons between the two forms of the BCB.

### Procedures

All patients with dementia had undergone general physical examination, laboratory examinations and CT or MRI of the head to exclude other causes of dementia. Patients and individuals from Control Group I were submitted to the neuropsychological battery used in the Reference Center for Cognitive Disturbances (CEREDIC – HCFMUSP), which consists of the application of the Clinical Dementia Rating Scale (CDR), Mini-Mental State Examination,^3,4^ Camcog,^16,17^ and phonemic Verbal Fluency tests (F.A.S. test). The Pfeffer Functional Activities Questionnaire,^5^ IQCODE (Informant Questionnaire of Cognitive Decline in the Elderly)^18,19^ and NPI^20^ were administered to informants.

In these two groups, the BCB was applied, only with a slight difference: the Clock Drawing test was performed after the delayed recall of the 10 objects. With this change, the interference period after the learning of the 10 objects was limited to the verbal fluency test. Two periods of time were measured: the time to accomplish the test from the beginning to the end of the delayed recall test, and the time from the beginning to the end of the recognition of the objects.

Besides the scores on each test, we introduced a new variable consisting of the difference between the delayed recall score and the learning test. The scores obtained using the mathematical formula were also investigated. Scores of the AD patients and both control groups were used for both the new variable and the mathematical formula.

This study was approved by the Ethics Committee of the Hospital das Clínicas of the University of São Paulo School of Medicine. All subjects were informed about the study prior to the evaluation, and written informed consent was given by the participant, or caregiver when necessary.

Descriptive statistics composed of mean and standard deviation values were used. The parameters of ROC (receiver operator characteristic) curve sensitivity, specificity and area under the curve (AUC) of both formats of the Battery were used to evaluate the efficiency of the abbreviated version versus the diagnosis obtained by the original version of the Brief Cognitive Battery. The value of significance accepted was 0.05 and the software package SPSS for Windows 14.0 (Chicago, IL) was used for the statistical analysis.

## Results

Demographic data and mean scores on the MMSE of patients and both control groups are shown in Table 1. Patients and controls from Group I did not differ in age, but had different educational levels and MMSE scores. Control Groups I and II did not differ on these characteristics.

**Table 1:** Means and standard deviations of demographic data and MMSE scores of 30 control individuals, 20 AD patients and 22 control individuals from a previous study.

	Control Group I (N=30)	AD (N=20)	p1	Control Group II (N=22)	p2
Age (years)	69.4 (7.32)	70.1 (10.35)	0.77	71.4 (7.26)	0.34
Schooling years	8.4 (5.08)	4.5 (4.01)	<0.01	8.36 (4.18)	0.96
MMSE	28.4 (1.45)	18.8 (4.54)	<0.01	27.77 (1.74)	0.14

Regarding the items of the BCB, patients and controls from Group I differed on all tests, except for the identification of the simple drawings task. The delayed recall continued to provide high accuracy in differentiating controls from dementia in AD (Table 2).

**Table 2:** ROC curves of patients and controls of Group I.

	AUC (95% CI)	Standard error^a^	Asymptotic significance^b^	Lower bound	Upper bound
Naming	0.765	0.082	0.003	0.604	0.92
Identification	0.559	0.090	0.507	0.382	0.73
Incidental memory	0.923	0.057	<0.001	0.00	1.0
Immediate memory	0.942	0.051	<0.001	0.00	1.0
Learning test	0.980	0.015	<0.001	0.00	1.0
Verbal fluency	0.946	0.034	<0.001	0.00	1.0
Delayed recall	0.970	0.020	<0.001	0.00	1.0
Recognition	0.955	0.037	<0.001	0.00	1.0
Clock-drawing	0.938	0.037	<0.001	0.83	1.0
MMSE	0.978	0.020	<0.001	0.00	1.0
Mathematical model	0.962	0.023	<0.001	0.88	1.0

a Under the nonparametric assumption; AUC: area under the curve;

b Null hypothesis: true area=0.5; MMSE: Mini-Mental State Examination.

The comparison between scores on the items of the BCB by control individuals from Group I and Group II, who underwent the abbreviated and original BCB, respectively, showed better scores on the delayed recall test in the abbreviated version (Group I). However, as the scores of Group I individuals were also better on immediate memory and showed a trend toward better performance on the learning test, we investigated the difference between delayed recall and learning test scores. This variable did not differ between groups (Table 3).

**Table 3:** Comparison between mean scores of control individuals (N=30) submitted to the abbreviated version and control individuals (N=22) submitted to the original version of the Brief Cognitive Battery.

	Control Group I (n=30)	Control Group II (n=22)	p
Mathematical formula	8.07 (11.46)	12.9 (15.81)	0.20
Identification	10 (0)	9.90 (0,30)	0.08
Naming	10 (0)	9.95 (0.21)	0.23
Incidental memory	5.67 (1.32)	5.27 (1)	0.06
Immediate memory	8.5 (1)	7.77 (1.3)	0.03
Learning test (lt)	8.4 (1.22)	8.54 (1.68)	0.06
Verbal Fluency	15.56 (4.19)	16 (3.91)	0.64
Delayed recall (dr)	8.4 (1.22)	7.54 (1.68)	0.03
Difference (dr-lt)	–0.66 (0.99)	–1 (1.15)	0.27
Clock-drawing test	8.7 (1.38)	8.13 (2)	0.21
Recognition	9.83 (0.46)	9.95 (0.21)	0.25

No difference was found between groups on the results of the mathematical formula.

In a previous study,^10^ the mean time to complete the full BCB was 487.7 seconds, with a standard deviation (SD) of 87.6 seconds and a median of 470 seconds. In the current study, a similar but slightly longer time period was needed: mean of 547.2 s (SD: 86s) for the *complete* Battery ( median time was 543s). For the abbreviated version, the mean was 391.8 s (SD 58.2 s) and the median 374.4 seconds. In other words, the mean time was reduced by 2 minutes and 37 seconds.

## Discussion

This study showed that the abbreviated version of the BCB was able to differentiate mild or moderate AD patients from controls while maintaining high accuracy, and had a shorter application time of approximately 2 and a half minutes in controls. The delayed recall test remained highly accurate in distinguishing AD patients from control individuals, showing that the reduction of the interference period between the learning and free delayed recall did not decrease the value of this test for reaching diagnosis. The mathematical formula also showed high accuracy in the differential diagnosis between AD and controls with the use of the abbreviated BCB.

Other brief batteries have been proposed for screening and for the diagnosis of dementia. Among them, the Mini-Cog,^21^ Memory Impairment Screen,^22^ the combination of word generation tasks and a delayed recall^23^ have been cited. The influence of educational level may be relevant in these tests.^10^ The Fuld Object Memory Evaluation (FOME) may be less influenced by educational level.^24^

The comparison between the two control groups showed a difference in the delayed recall test, with better results found in individuals from Group I, who were evaluated with the abbreviated BCB. This could be interpreted as evidence that the delayed recall of the abbreviated BCD was easier than its counterpart in the original BCB. However, the performance of the control individuals from Group I was better in immediate memory and they also showed a trend towards better performance in incidental and learning tests, which may have been responsible for their better scores on the delayed recall test. These differences could be explained by the low number of individuals in the samples. Comparison of delayed recall and learning scores for both control groups revealed no differences. The absence of difference between the delayed recall and learning scores lends support to our hypothesis that the abbreviated BCB is reliable.

No differences on the mathematical formula were seen between the two control groups, further supporting the potential value of the abbreviated BCB.

Our study however, had several limitations: the low number of AD patients and control individuals, the presence of cases with moderate dementia, and the lower level of education of AD patients compared to controls. Further studies should be conducted to overcome these limitations.

The possibility of using the BCB without the clock drawing test, thereby simplifying its application and allowing application within an average time of 6 and a half minutes, makes the abbreviated BCB a potentially valuable tool for screening dementia of AD in population studies, as well as in clinical practice among developing countries, whose populations have a heterogeneous educational background.

## Figures and Tables

**Figure 1:** The mathematical formula with high accuracy for the diagnosis of Alzheimer's disease using the Brief Cognitive Battery^11^.

Score = 100 ×	exp (8.518 – (0.680*dr) – (0.475*learn) – (0.186*vf) + (0.119*school))
	1 + exp (8.518 – (0.680*dr) – (0.475*learn) – (0.186*vf) + (0.119*school))

dr, delayed recall score; learn, learning test score; vf, category verbal fluency score; school, years of schooling.
